# Apurinic/apyrimidinic endonuclease 1 is a key modulator of aluminum-induced neuroinflammation

**DOI:** 10.1186/1471-2202-14-26

**Published:** 2013-03-11

**Authors:** Amira Zaky, Bassma Mohammad, Marie Moftah, Kamal M Kandeel, Ahmad R Bassiouny

**Affiliations:** 1 Department of Biochemistry, Faculty of Science, Alexandria University, Alexandria, Egypt; 2 Department of Zoology, Faculty of Science, Alexandria University, Alexandria, Egypt

**Keywords:** Neuroinflammation, Apurinic / apyrimidinic endonuclease1 (APE1), Aluminum chloride (AlCl_3_), Resveratrol (resv)

## Abstract

**Background:**

Chronic administration of Aluminum is proposed as an environmental factor that may affect several enzymes and other biomolecules related to neurotoxicity and Alzheimer's disease (AD). APE1 a multifunctional protein, functions in DNA repair and plays a key role in cell survival versus cell death upon stimulation with cytotoxic agent, making it an attractive emerging therapeutic target. The promising protective effect of resveratrol (resv), which is known to exert potent anti-inflammatory effects on neurotoxicity induced by aluminum chloride (AlCl_3_), may be derived from its own antioxidant properties. In the present work we investigated the modulation of APE1 expression during AlCl_3_-induced neuroinflammation (25 mg/Kg body weight by oral gavages) in experimental rats. We tested the hypothesis that a reactive oxygen species (ROS)-scavenger, resveratrol at 0.5 mg/kg bodyweight, which is known to exert potent anti-inflammatory effects, would attenuate central inflammation and modulate APE1 expression in AlCl_3_-fed rats. Neuroinflammation-induced genes including β-secretase (BACE), amyloid-β precursor protein (APP), presenilin 2 (PSEN-2) and sirt-2 were determined by RT-PCR. APE1 is determined at mRNA and protein levels and confirmed by immunohistochemistry. The expression of pro-inflammatory cytokines (TNF-α, IL6) and iNOS by the rat brain extract were measured by RT-PCR.

**Result:**

Our results indicate that resveratrol may attenuate AlCl_3_-induced direct neuroinflammation in rats, and its mechanisms are, at least partly, due to maintaining high APE1 level. Resveratrol co-administration with aluminum chloride exerted more protective effect than pre-administration or treatment of induced rats. A significant elevation of APE1 at both mRNA and protein levels was observed in addition to a marked reduction in β-secretase and amyloid-β. We found that AlCl_3_ stimulated the expression of TNF-α, IL-6, and iNOS in rat brain in which NF-κB was involved. Resveratrol inhibited AlCl_3_-induced expression and release of TNF-α, IL-6, and iNOS in rat brain.

**Conclusions:**

These findings establish a role for APE1 as a master regulator of AlCl_3_ dependent inflammatory responses in rat brain. In addition, there was an ameliorative change with resveratrol against AlCl_3_-induced neurotoxicity. These results suggest that rat brain cells produce pro-inflammatory cytokines in response to AlCl_3_ in a similar pattern, and further suggest that resveratrol exerts anti-inflammatory effects in rat brain, at least partly, by inhibiting different pro-inflammatory cytokines and key signaling molecules. It might be a potential agent for treatment of neuroinflammation-related diseases, such as AD.

## Background

Aluminum is a well-documented neurotoxin that enhances neuroinflammatory events in the brain by different mechanisms. Aluminum exacerbates oxidative stress, amyloid beta (Aβ) deposition, and plaque formation in the brain of transgenic mice that overexpress amyloid beta (A4) precursor protein (APP) [1]. Both Aβ and aluminum are able to potentiate reactive oxygen species (ROS) formation that will lead to genotoxicity and DNA damage. The mammalian ap-endonuclease, APE1/ref-1, is a ubiquitous and remarkably multifunctional protein. It plays a central role in the base excision repair (BER) pathway for damaged bases and DNA single-strand breaks induced by ROS and alkylating agents [2]. APE1 was independently identified as a reductive activator factor and named redox effector factor 1 (Ref-1) [3]. A third and distinct function of APE1 as a trans-acting factor was also discovered [4,5]. Several studies showed that global cerebral ischemia or traumatic brain injury or cold injury-induced brain trauma [6] induced oxidative stress decreases APE1 expression in the hippocampus and is associated with neuronal apoptosis in rats [7,8]. This specific inhibition of APE1 expression may affect the extent of apoptosis after ischemia. Consistently, overexpression of WT APE1 in hippocampal and sensory cells reduced neuronal death [9]. Moreover a very recent study by Mantha et. al., [10] indicated that APE1/Ref-1 exerts neuro-protective role via its association with different intracellular proteins in Aβ (25–35)-treated rat pheochromocytoma, PC12 and SH-SY5Y cell lines, which could modulate their cellular functions during Aβ-mediated neurotoxicity.

The polyphenolic compound resveratrol (3,4',5-trihydroxy-trans-stilbene) is a naturally occurring phytochemical which has been found in a large number of plant species that are components of human diet, including mulberries, peanuts, grapes and red wine. Its physiological function is thought to serve as phytoalexin protecting plants against environmental stress or pathogen attack. Accumulating evidence suggests that resveratrol may exert a protective effect in the CNS under pathological conditions, and that resveratrol is associated with reduced risks of cardiovascular disease, cancer, diabetes and AD [11-13].

Resveratrol has been found to exert protective effects against neuroinflammation in both in vivo and in vitro studies. These activities of resveratrol appear to target activated microglia, resulting in the reduction of pro-inflammatory factors through the modulation of signal transduction pathways. Activated microglia and astrocytes, the main glial cell type, serve immune surveillance functions and are involved in maintaining CNS homeostasis. They also respond promptly to injury and regulate neuroinflammatory events [14,15]. Over-activation of glial cells and release of pro-inflammatory cytokines may lead to neuronal death [16,17], causing neuropathological changes in CNS diseases such as multiple sclerosis [18], Parkinson's disease [19] and Alzheimer's disease [20]. Therefore, limiting inflammatory cytokine production by activated microglia and astrocytes should be beneficial for prevention of neuroinflammation and neurodegeneration.

One of the potential mechanisms for resveratrol-mediated neuroprotection is activation of the Sirt1 pathway, which in turn suppresses the activation of the NF-κB signaling pathway [21]. The overall effects are to reduce pro-inflammatory mediators, eventually producing neuroprotection. Sirt1could also protect neurons against microglia-dependent Aβ toxicity via the suppression of NF-κB pathway [21].

Yamamori et. al., [22] reported that APE1 is a target of the Sirt1 protein deacetylase. Sirt1 associates with APE1, and this association is increased with genotoxic stress and cell vulnerability is rescued by overexpression of APE1. Activation of Sirt1 with resveratrol promotes binding of APE1 to the BER protein X-ray cross-complementing-1 (XRCC1), while inhibition of Sirt1 decreases this interaction, which suggests that Sirt1 plays a vital role in maintaining genomic integrity through regulation of the BER pathway.

In the present study we show for the first time the involvement of APE1 modulation in resveratrol-mediated therapeutic and/or protective activity against aluminum chloride-induced neurotoxicity in rats. We examined also the expression of pro-inflammatory cytokines (TNF-α, and IL-6) and of iNOS in brain extract in response to AlCl_3_ exposure, as well as the NF-κB signaling pathway.

## Methods

### Animals and neuroinflammation induction

**Figure 1:**
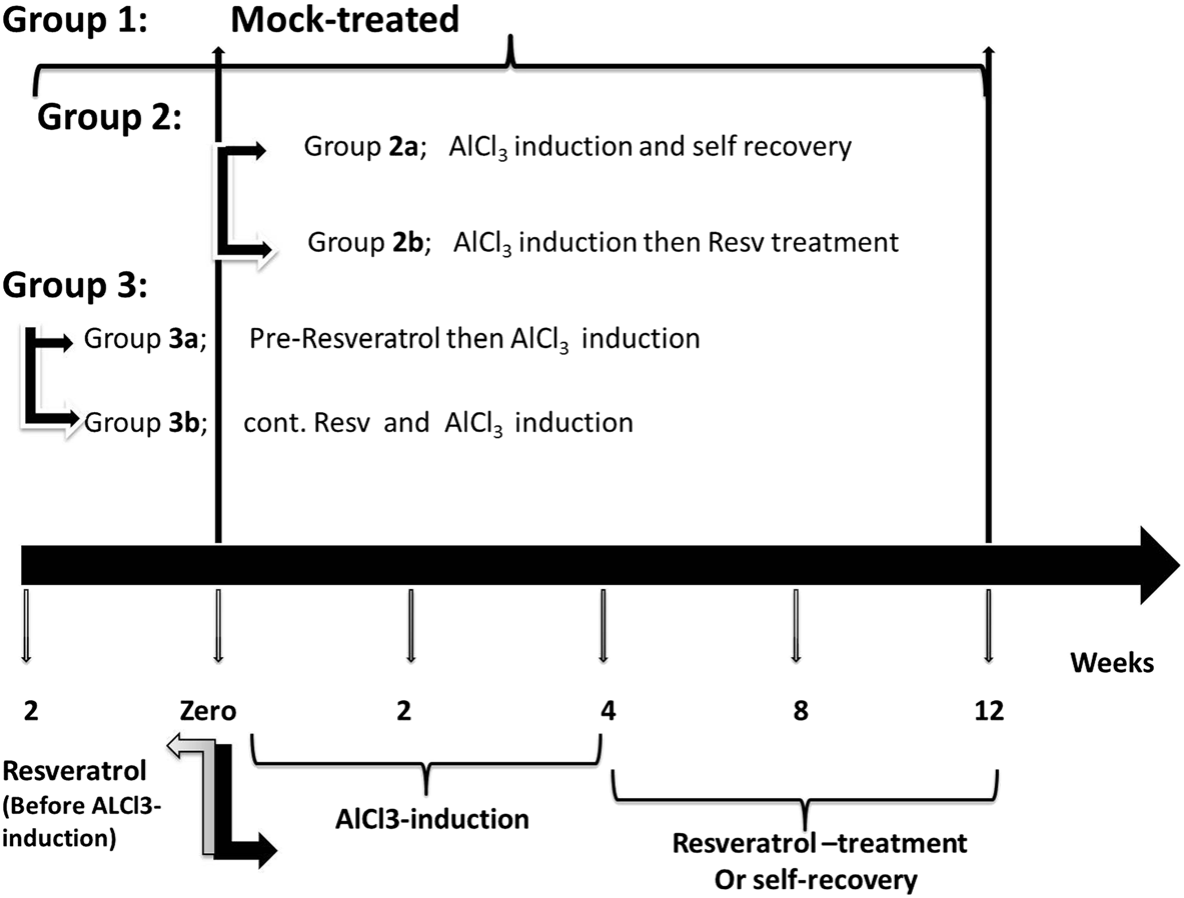
Experimental protocol design.

Total of forty male adult Wistar rats (120–190 g) were supplied and maintained at Medical Research Institute in which the principles of laboratory animal care were followed in all protocols and were approved by ethics committee of animal research facility. Rats were maintained under controlled temperature (25°C) and constant photoperiodic conditions (12:12-h daylight/darkness). The dams had free access to water and standard commercial chow. Neuroinflammation was induced using AlCl_3_.6H_2_O. Animals were divided into three major groups as illustrated in Figure 1. Induced rats received AlCl_3_.6H_2_O (25 mg/ kg) daily for duration of one month by oral gavages. Resveratrol was administered by gavage in the form of resveratrol and green tea complex (0.5 mg/ kg).

#### Samples collection

Three rats from each group were sacrificed by cervical dislocation at time intervals 2 and 4 weeks of AlCl_3_ administration and at weeks 8 and 12 of resveratrol treatment or self-recovery. Brains were rapidly removed from the skulls and dissected into different regions. Parts of the Cortex and hippocampus regions were removed for histochemical studies by fixation with10% buffered-saline formalin. The remaining sections were stored at −80°C for extraction of total RNA and enzyme assays.

### Assay of oxidative stress-related markers

#### Reduced glutathione (GSH)

Part of the brain tissue (10% w/v) was washed with saline solution minced and homogenized in ice cooled buffer 1.15% KCl, 0.01 M sodium phosphate buffer pH 7.4. Concentration of GSH was determined as described by Ellman [23].

#### Lipid peroxidation

Part of the brain tissue (10% w/v) was washed with saline solution, minced and homogenized in ice-cooled 50 mM potassium phosphate buffer pH 7.5. Level of lipid peroxidation was determined according to Ohkawa e.t al., [24] method.

#### Glutathione-s-transferase (GST) activity

Brain tissue was homogenized in ice-cooled buffer (100 mM potassium phosphate, pH 7.0 containing 2 mM EDTA) per gram tissue. GST was assayed according to Habig et. al., method [25].

#### Catalase activity

Part of brain tissue was homogenized in ice-cooled homogenization buffer (50 mM potassium phosphate, pH 7.4, 1 mM EDTA and 1 ml/L Triton X-100). Catalase activity was assayed according to Aebi procedure [26].

#### Aspartate amino transferase (AST) Activity

AST activity assay in brain tissues was preformed according to the method described by Reitman and Frankel [27] with some modifications. Briefly part of mid-brain tissue was dissected, washed in ice-cooled saline and homogenized in total protein extraction buffer (10 mM HEPES, 350 mM sucrose, 5 mM EDTA, pH 7.4, 1% of Triton-X100, and protease inhibitor cocktail) then centrifuged at 4,000 rpm for 15 min at 4°C. Ten microlitters of the supernatant were used for AST activity assay as described before [27].

### Estimation of amyloid beta 40 (Aβ 40):-

This quantification was done using commercially available rat amyloid beta peptide 1–40 (Aβ1-40) ELISA Kit (Cusabio, cat# CSB-E08302r) and according to manufacturer's instruction with some modifications. Briefly brain tissues of experimental animals were isolated at the end of each experimental phase, washed in ice-cooled saline and homogenized in extraction buffer containing 10 mM HEPES, 350 mM sucrose, 5 mM EDTA, pH 7.4, 1% of Triton-X100, and protease inhibitor cocktail. The homogenates were analyzed and Aβ-40 was calculated as (pg/ml/g. tissue).

### Isolation of Total RNA and semi qRT-PCR analysis

**Figure 2:**
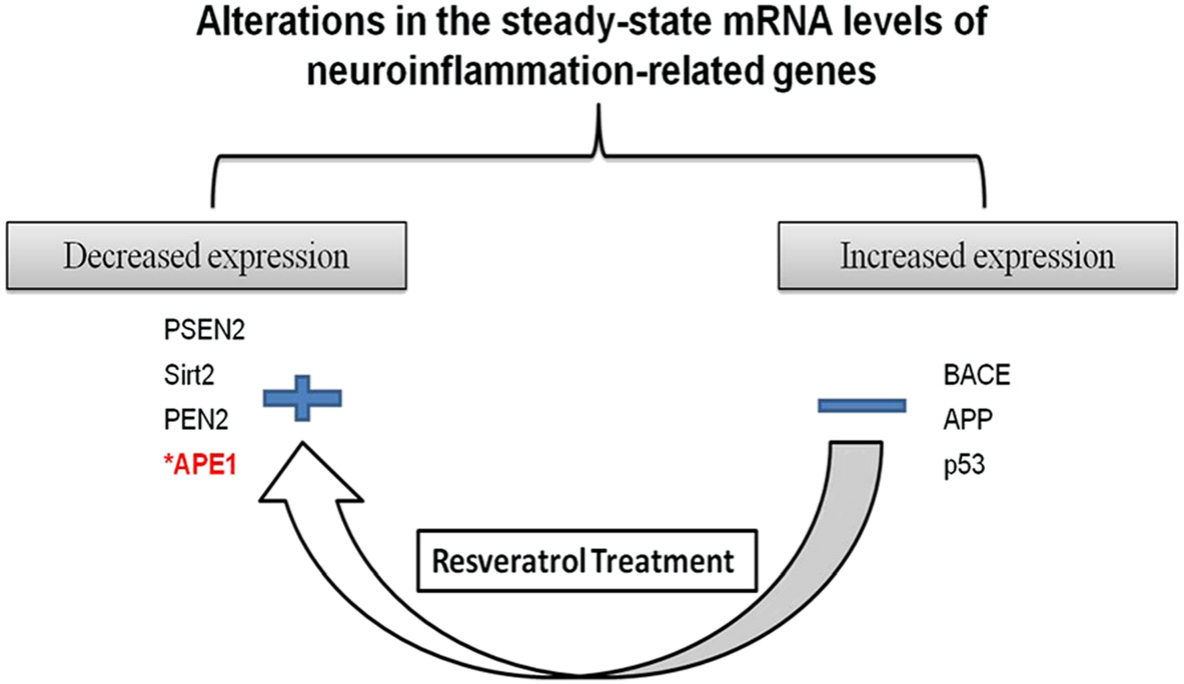
**Outline of the neuroinflammation-induced genes in AlCl**_**3**_**-induced versus resveratrol-treated state.**

Total RNA was extracted from frozen brain tissues according to the method of Chomczynski and Sacchi procedure [28]. Alteration in the steady–state mRNA levels of genes relevant to neuroinflammation pathogenesis (Figure 2) was determined using semi-quantitative reverse transcriptase PCR analysis. Using one-step RT-PCR (RT/PCR Master Mix Gold Beads, BIORON) reaction, the cDNA was synthesized and used for amplification of target gene(s) primers sequences: Amyloid beta (A4) precursor protein (APP)-F: AGAGGTCTACCCTGAACTGC, R: ATCGCTTACAAACTCACCAAC- 154 bp; beta secretase (BACE)-F: CGGGAGTGGTATTATGAAGTG, R: AGGATGGTGATGCGGAAG, 320 bp [29]; presenilin 2 (PSEN2) F:GAGCAGAGCCAAATCAAAGG**,**R-GGGAGAAAGAACAGCTCGTG,188 bp; Sirt2 F:ACCTTCCTTCAGTCCCGTTT,R: AAGGGTTCACAGTGGTGGAG,173 bp; TNFαF:ATGAGCACAGAAAGCATGATCCGCG,R:CCCTTCACAGAGCAATGACTCCAAA; IL-6-F: GATGCTACCAAACTGGATATAATC, R:GGTCCTTAGCCACTCCTTCTGTG; iNOS-F: TGGGAATGGAGACTGTCCCAG, R:GGGATCTGAATGTGATGTTTG; β-actin-F: TGTGATGGTGGGAATGGGTCAG, R: TTTGATGTCACGCACGATTTCC.

### qRT-PCR assay

Real time-PCR (qRT-PCR) was used to measure the mRNA expression levels of APE1 gene. CDNA was synthesized by High-Capacity cDNA Reverse Transcription Kit according to the manufacture`s protocol. APE1 Primers sequence: 5’-GCTTGGATTGGGTAAAGGA-3’ and 3’TTCTTTGTCTGATGGAGCTG-5’; GAPDH primers: 5’-GTATGACTCTACCCACGGCAAGT-3’and 5’-TCTCGCTCCTGGAAGATGGT-3’. APE1 was normalized to GAPDH and the fold difference calculated using the equation 2^−ΔΔCt^ as described before [30].

### Preparation of total and nuclear extracts

Total cell extracts were prepared by homogenization of 50–100 mg of tissue in lysis buffer (50 mM Tris–HCl, pH 7.5, 150 mM NaCl, 1 mM EDTA, 1% TritonX-100, and protease inhibitor cocktail). Nuclear extract was prepared as described by Schreiber et. al., [31].

### Western blotting

Western blots were performed as described previously by Burnette [32], APE1 and NF-κB immunoblots were performed on prepared total and nuclear cell extracts respectively. Primary antibody to APE1 (sc-17774), NF-κB (NB100-2176) and β-actin (sc-81178) were used. Antibody binding was detected following appropriate secondary antibody using chemiluminescence detection, and equal loading was confirmed by probing with β-Actin monoclonal antibody.

### Statistical analysis

All experiments were performed in duplicate or triplicate independently and typical graphs are presented in some cases data are expressed as Mean± SD. Data were analyzed by student’s t- test and difference was considered significant from control when p < 0.01. ANOVA test was used to compare the statistical difference between groups. The results are considered significant when p < 0.05.

## Results

### Effect of resveratrol administration on brain`s total anti-oxidant capacity, tissue AST activity and Aβ 40 level

**Figure 3:**
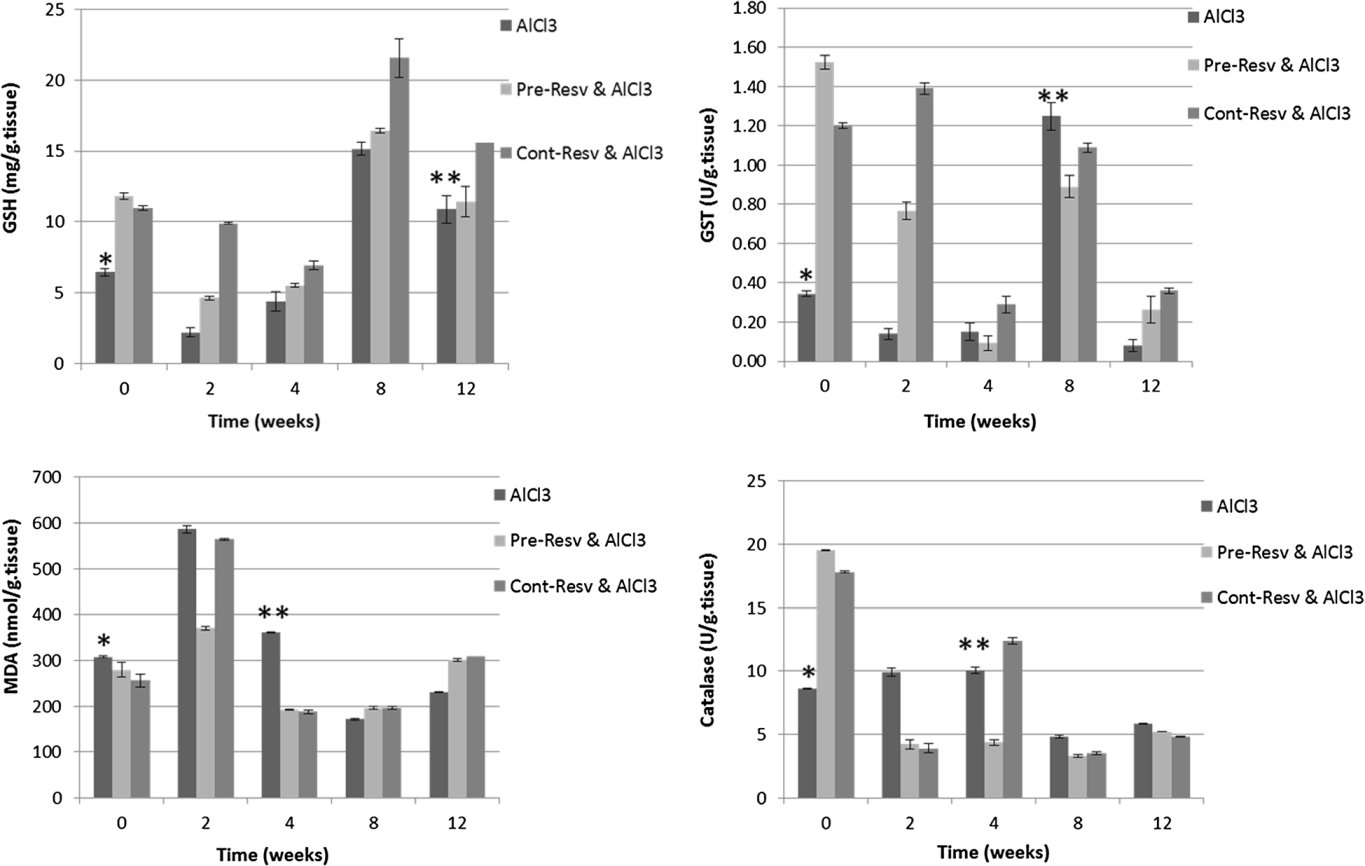
**Total anti-oxidant capacity in AlCl**_**3**_**-induced versus resv-treated rats.** A significant reduction in GSH contents as well as GST and catalase activities during the four weeks of AlCl3 administration while MDA level was increased significantly (*p < 0.01) compared to control. Pre-and continuous resveratrol administration ameliorated toxicity by maintaining high GSH level and enzymes activities. Also we show that resv-treated rats post AlCl_3_ induction improved the total antioxidant capacity in both pre-and cont- compared to self-recovery group (**p < 0.05).

Oral AlCl_3_ administration for four weeks was tolerated by the majority of rats, with less than 3% mortality. Initially all rats lost few grams of the body weight during the first 10 days of the study. However, rats in all groups re-gained weight and continued to grow normally for the duration of the study. Analysis of anti-oxidant capacity represented by GSH and MDA levels as well as catalase and GST activities revealed that AlCl_3_- induced significant elevation in MDA along with marked reduction in GSH contents, GST and catalase activities at weeks 2 and 4 compared to mock-treated group at week zero (p < 0.01, Figure 1A-D). Pre- and cont- resveratrol administered groups 3a & b, showed higher anti-oxidant capacity compared to self-recovery (p<0.05, Figure 3). However, continuous resveratrol administration is shown to be more protective compared to both self-recovery and pre- groups (p < 0.01, p < 0.05, Figure 3).

**Figure 4:**
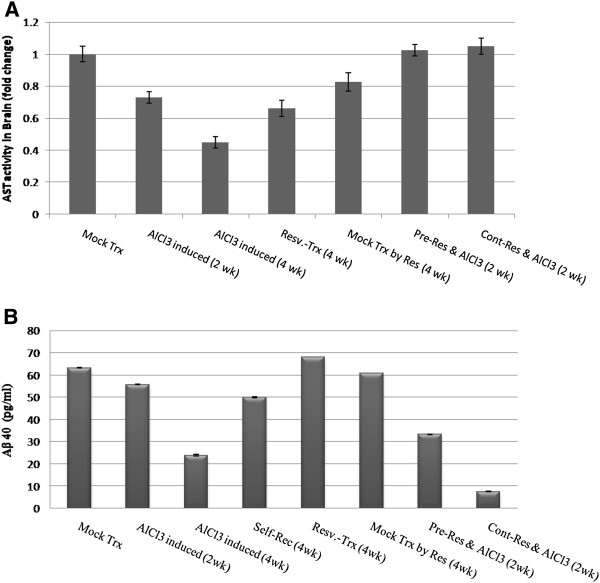
**Brain AST activity and Aβ 40 level. A**- alterations in brain tissues AST activities (mid brain) indicating significant reduction during the four weeks of AlCl_3_ administration compared to control (P < 0.01). Pre- and continuous resveratrol administration ameliorated toxicity by maintaining tissue integrity and hence elevated AST activity. **B**- Aβ 40 concentration significant reduced in AlCl_3_-induced versus resv-treated rats. Pre-and continuous resveratrol significantly (p < 0.01) maintained high Aβ 40 level upon AlCl_3_ administration.

AlCl_3_-induced neurotoxicity was further assessed by measuring tissue AST activity, which is important enzyme in brain that is strongly related to amino acid homeostasis. The results indicated significant (p < 0.01) reduction in AST activity in the induced rats. While resveratrol administration markedly improved (p < 0.01) AST activity in different experimental groups as represented in Figure 4A.

Furthermore by detecting the level of Aβ 40, which is considered target for Alzheimer's therapy, in experimental rats` brain we found that resveratrol-induced significant elevation (p < 0.01) in its level compared to AlCl_3_-induced rats (Figure 4B).

### Profile of neuroinflammation-related genes expression in induced versus treated rats

**Figure 5:**
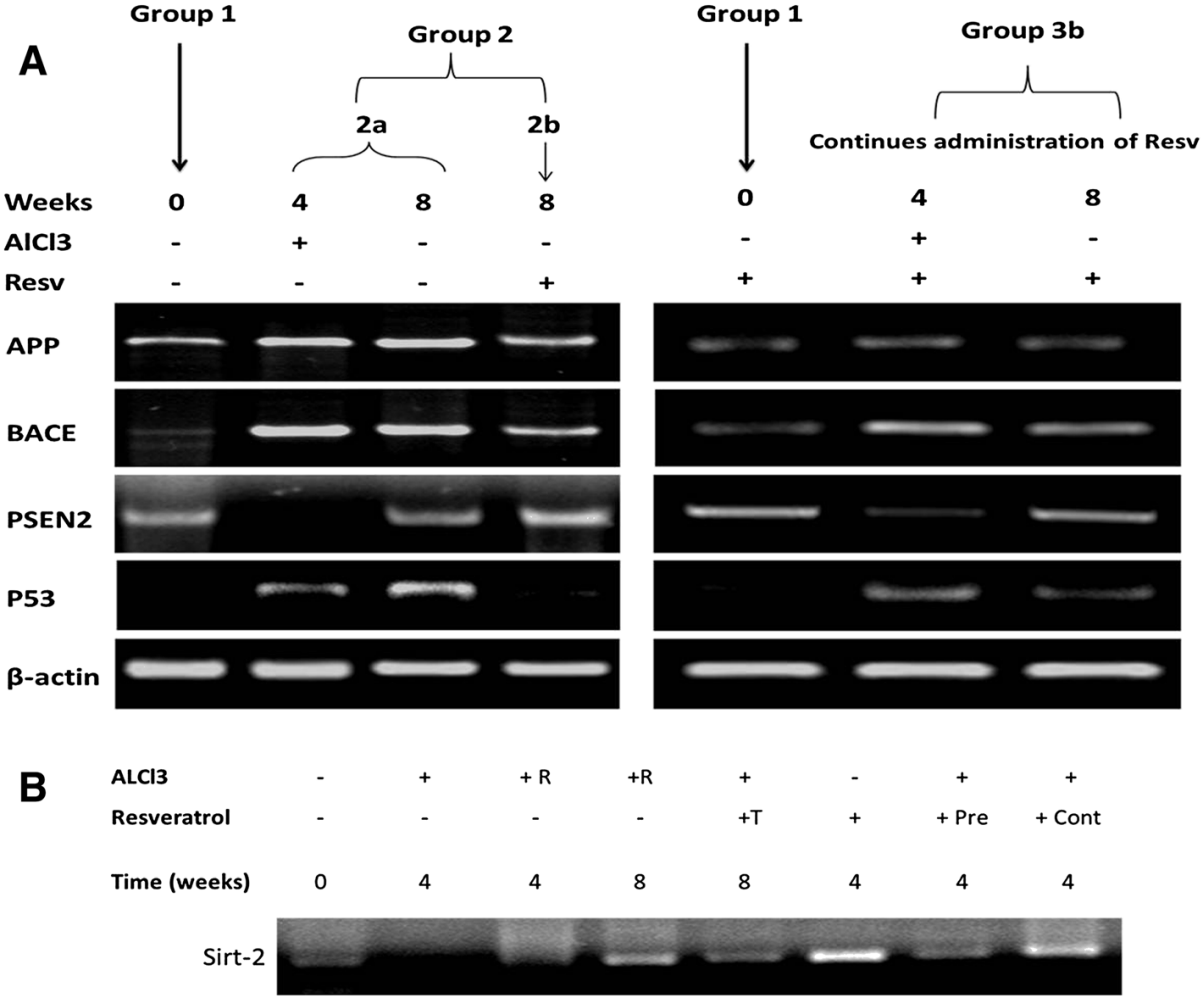
**Expression profiles of neuro-inflammation related genes.** Semi-quantitative RT-PCR was used to detect mRNA levels of APP, BACE, PSEN2, p53 and sirt-2. **A-**Our results indicate that AlCl_3_ administration induces elevation in APP, BAC and p53 levels at week four (group2a), while resv treatment (group 2b) caused marked reduction in all parameters mRNA levels compared to self-recovery group (2a at week 8). Co-administration of resv during AlCl3 induction and for four weeks later ameliorated neurotoxicity by reducing mRNA levels of pro-inflammatory genes as shown in group3b at weeks 4 and 8. Moreover PSEN2 expression is induced in resv treated groups 2b and 3b. **B-** Resveratrol induces Sirt-2 in mock-treated group and resveratrol treatment of AlCl_3_-induced group induces sirt-2 expression as well.

Alterations in the expression of neuroinflammation related markers (Figure 2), post AlCl_3_ feeding at weeks 4 and 8 (groups 2a, b and 3b) was tested by semi-quantitative PCR. Strong expression of APP, BACE and p53 were observed in the induced group that received no resveratrol during the course of induction compared to lower expression in the continuously administered group at week 4 (Figure 5A). A decrease in the mRNA of all pro-inflammatory mediators was observed in resv-treated rats compared to self-recovery group (2a and 2b respectively). Interestingly, we found that resv continuous administration exerted more protection through marked inhibition of inflammatory markers (Figure 5A, group 3b at weeks 4 and 8) and induction of PSEN2 expression in both groups. By testing sirt-2 expression we found that resveratrol treatment induced sirt-2 expression in pre-, cont- and treated groups which indicate that sirt-2 is involved during neuronal recovery from injury (Figure 5B).

**Figure 6:**
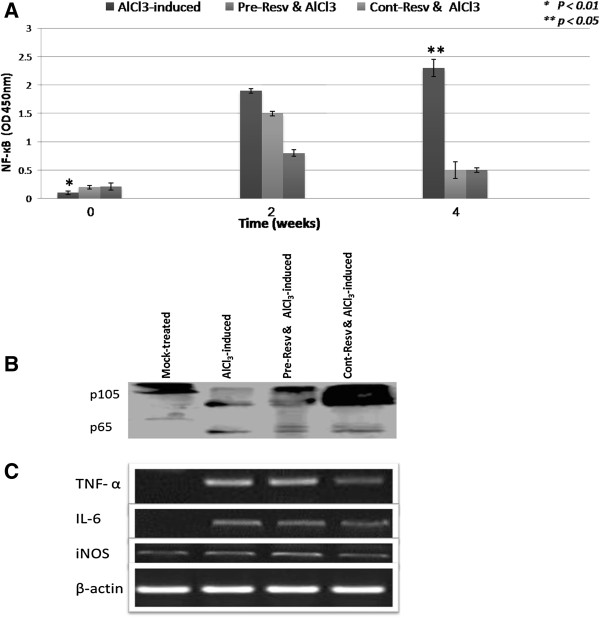
**NF-κB, cytokines and iNOS profile in different experimental groups.** A & B) Change in NF-κB activity and protein levels were detected in induced versus resveratrol administered groups. We show that AlCl_3_ administration induced significantly (*p < 0.01) NF-κB activity with more p65 dissociation from the p105 complex as represented in the western blot. On the contrary co-administration of resveratrol with AlCl_3_ in both pre- and con- groups significantly decreased (**p < 0.05) NF-κB activity as well as p105 complex dissociation (B). Resveratrol inhibits AlCl_3_-induced TNF-α, IL-6 cytokine and iNOS expression in rat brain (C).

Because activation of Sirt1 pathway, which in turn suppresses the activation of the NF-κB signaling cascade, is one of resveratrol well documented mechanism for reduction of proinflammatory mediators, therefore we measured NF-κB transcription factor activity as well as protein level. NF-κB activity as well as p105 complex dissociation were elevated significantly in AlCl_3_-induced group in time-dependent manner compared to mock-treated group (p < 0.01, Figure 6 A & B). On the contrary, pre- and cont- resveratrol administered groups showed significant reduction in NF-κB activity as well as p65 dissociation from p105 complex compared to induced group (p < 0.05, Figure 6 A & B). Furthermore we examined the effect of resveratrol on AlCl_3_ -induced proinflammatory cytokine expression in rat brain homogenate. As shown in Figure 6C, AlCl_3_ markedly increased TNF-α, IL-6 mRNA expression in rat brain homogenates. Continuous resv administration exerted more inhibitory effect on cytokines and iNOS expression than pre-administration (Figure 6C).

### APE1 as a novel molecular target in resveratrol-mediated neuroprotection

**Figure 7:**
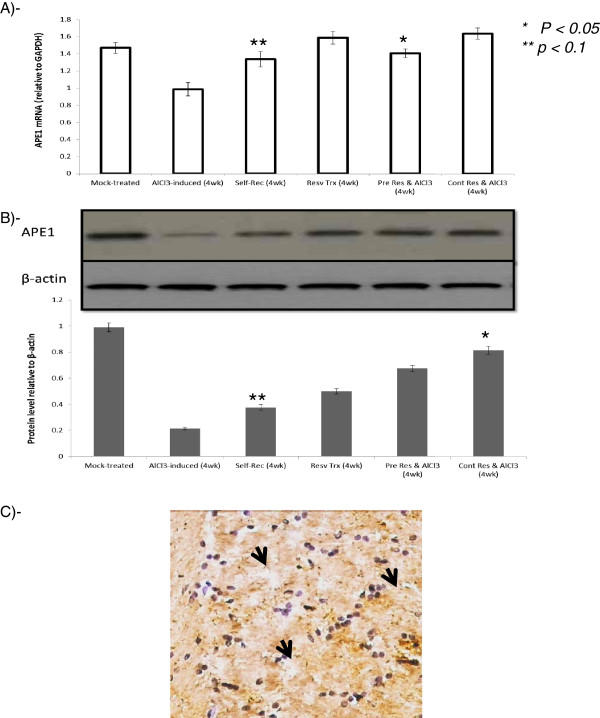
**Changes in APE1 mRNA and protein levels.A-**mRNA was determined using qRT-PCR. A significant reduction (p < 0.01) in group 2a at 4 weeks of induction was observed. APE1 mRNA was elevated during self-recovery (group 2a at week 8) as well as during the four weeks of resveratrol treatment. Pre- and cont- resveratrol administration maintained significantly elevated (p < 0.01) APE1 compared to induced group. **B-** APE1 protein profile shows the same pattern and change in parallel to mRNA as indicated in all groups. **C-**Immunohistochemical analysis of continuously resveratrol administered group indicated strong cytoplasmic expression of APE1. * indicates significance from control at p < 0.01, while ** indicates significance between groups at p < 0.05.

APE1 was detected at both levels mRNA using qRT-PCR and protein using western blotting as well as by immunohistochemistry. AlCl_3_ oral administration for 4 weeks significantly reduced APE1 both at mRNA and protein levels versus mock-treated group (p < 0.01, Figure 7). Although resveratrol treatment was observed to act through induction of APE1 expression, we also observed that during self-recovery of injured- rats, brain APE1 level is elevated significantly (p <0.01, p < 0.05, Figure 7A-B).Very interestingly, pre- and cont-resveratrol administration with AlCl_3_ maintained high APE1 mRNA and protein and exerted more protective effect than post-lesion treatment (p < 0.01, p < 0.05, Figure 7A-B). Moreover immunohistochemical investigation showed strong cytoplasmic labeling of APE1 in brain sections of continuously administered group that received resv during AlCl_3_ induction (Figure 7C).

## Discussion

Oxidative stress and extensive DNA damage has been reported as contributing factor in different diseases including neuronal degeneration. The arguments on the role of Aluminum (Al)-induced oxidative stress and mediated neuronal loss may help in understanding the role of Al in Alzheimer’s disease (AD). Neurons appear to be particularly vulnerable to free radicals. Al, which is a stress-inducing agent in endoplasmic reticulum, has been shown to activate the expression of various genes that are important in growth arrest and DNA damage induction, and NF-κB, which initiates apoptosis [33].

To explore the mechanism of resveratrol on the attenuation of AlCl_3_-induced neuroinflammation, the expression of TNF-α, COX-2 and APP protein expression was detected by RT-PCR, as well as induction of NF-κB in the rat brain by western blot.

Our findings are in agreement with Wu Z et. al., report [34] and clearly show that resveratrol attenuates AlCl_3_-induced neuroinflammation. We demonstrate that continued resveratrol administration during the course of induction exerts more protective effect than pre- or after induction administration. By detecting total lipid peroxidation and glutathione contents in rat`s brain, a significant reduction in lipid peroxidation as well as a significant increase in brain glutathione contents were observed in all resveratrol treated groups versus induced rats.

NF-κB which considered an important transcription factor in inflammatory responses can regulate the production of various pro-inflammatory factors [35]. In the resting conditions, NF-κB is sequestered in the cytoplasm by binding to its inhibitors IκBs. In response to inflammatory stimuli, IκBs are rapidly phosphorylated and then degraded via IKK complex, followed by the release of free NF-κB dimers (p50 and p65) and subsequent translocation to the nucleus and thus regulating the expression of target genes. Sirt-1 upregulation could protect neurons against microglia-dependent Aβ toxicity via the suppression of NF-κB pathway [36]. Therefore, we investigated the effect of resv as therapeutic and/or prophylactic agent on NF-κB activation and p105 complex stability. We found that resv co-administration during AlCl_3_-feeding resulted in a significant reduction in both NF-κB activity and p105 complex dissociation compared to resv-treated group and resv continues administration inhibited AlCl3- mediated NF-κB activation and complex dissociation. To further explore the resveratrol inhibitory effect on AlCl_3_- induced brain toxicity, we detected the mRNA level of neuroinflammation-regulated genes including APP, β-secretase (BAC), p53, Presenilin 2 (PSEN2), Sirt2, as well as APE1 as suggested new target for resveratrol-mediated neuro-protection. It has been shown that the abnormal processing of APP by β and γ-secretase enzymes is a key event in the development of Alzheimer's disease (AD) neuropathology, resulting in an increase in the generation of the 42 amino acid form of Aβ peptide which aggregates to form the insoluble amyloid plaques [37].

The γ-secretase complex has not yet been fully characterized but minimally consists of four individual proteins including presenilin (PSEN) [38]. Here we show that continued oral administration of resveratrol markedly repressed AlCl_3_-induced APP mRNA expression and decreased tissue Aβ 40 level through down regulation of BAC expression and also by reducing γ-secretase activity probably through down-regulation of PSEN2 as a regulatory subunit. Resveratrol-treated rats showed higher brain Aβ 40 levels compared to induced group. Jayadev et. al., [39] report demonstrated that PSEN2 regulates CNS innate immunity through the finding that PSEN2 is the predominant γ-secretase in microglia and modulates release of proinflammatory cytokines, therefore PSEN2 may participate in a negative feedback loop regulating inflammatory behavior in microglia. In our model PSEN2 expression as a neuro-protective marker found to be induced by resveratrol administration either as treatment or protective agent, which clarify that PSEN2 is one of the resv-activated genes during neural regeneration. It has been reported that a redundancy of functions may exist between sirt-1 and sirt-2, and that sirt-1 and sirt-2 cooperate to deacetylate the tumor suppressor protein p53 to attenuate cell death [40]. Moreover many reports highlighted the role of sirt1-sir2 as a target for resv-mediated action. Here we show that resv-induces the expression of sirt-2 which considered mitotic protein that promotes cell survival. We found that in mock-treated rats, administered only resv as a positive control for four weeks, a strong sirt-2 expression was detected compared to empty vehicle control group. Also resv treatment, pre- or cont- administration induced sirt-2 expression, suggesting a pivotal role for cytoplasmic protein sirt-2 during neural cells regeneration or protection. In neurons, base excision repair (BER) is the predominant mechanism for repair of oxidative DNA lesions. In addition it has been reported that Aβ level differentially modulates APE1 expression which may contribute to selective neuronal vulnerability in Alzheimer’s disease [41]. The inhibition of Sirt1 signaling by AlCl_3_ is partially responsible for the activation of NF-κB pathways and subsequent generation of TNF-α in Kupffer cells and macrophages [42]. Therefore, it should be quite interesting to investigate whether activation of Sirt1 signaling also contributes to the inhibitory effect of resveratrol on NF-κB activation by AlCl_3_ in rat brain cells. AlCl_3_ is capable of inducing production of pro-inflammatory cytokines and NO in treated rat brain, probably by both microglia and astrocytes [43]. In agreement with our finding, aluminum causes oxidative damage as a pro-oxidant, both on its own and in synergy with iron. Aluminum also competes with, and substitutes for, essential metals—primarily Mg^2+^, iron and Ca^2+^ ions—in or on proteins and their co-factors. It was hypothesized that intra-neuronal aluminum may interfere with Ca^2+^ metabolism in the aged brain [44]. Our study shows that AlCl_3_ significantly induces the expression and production of pro-inflammatory cytokines (TNF-α, IL-6,), and enhances the expression of iNOS most probably by immuno-competent and phagocytic cells in CNS.

**Figure 8:**
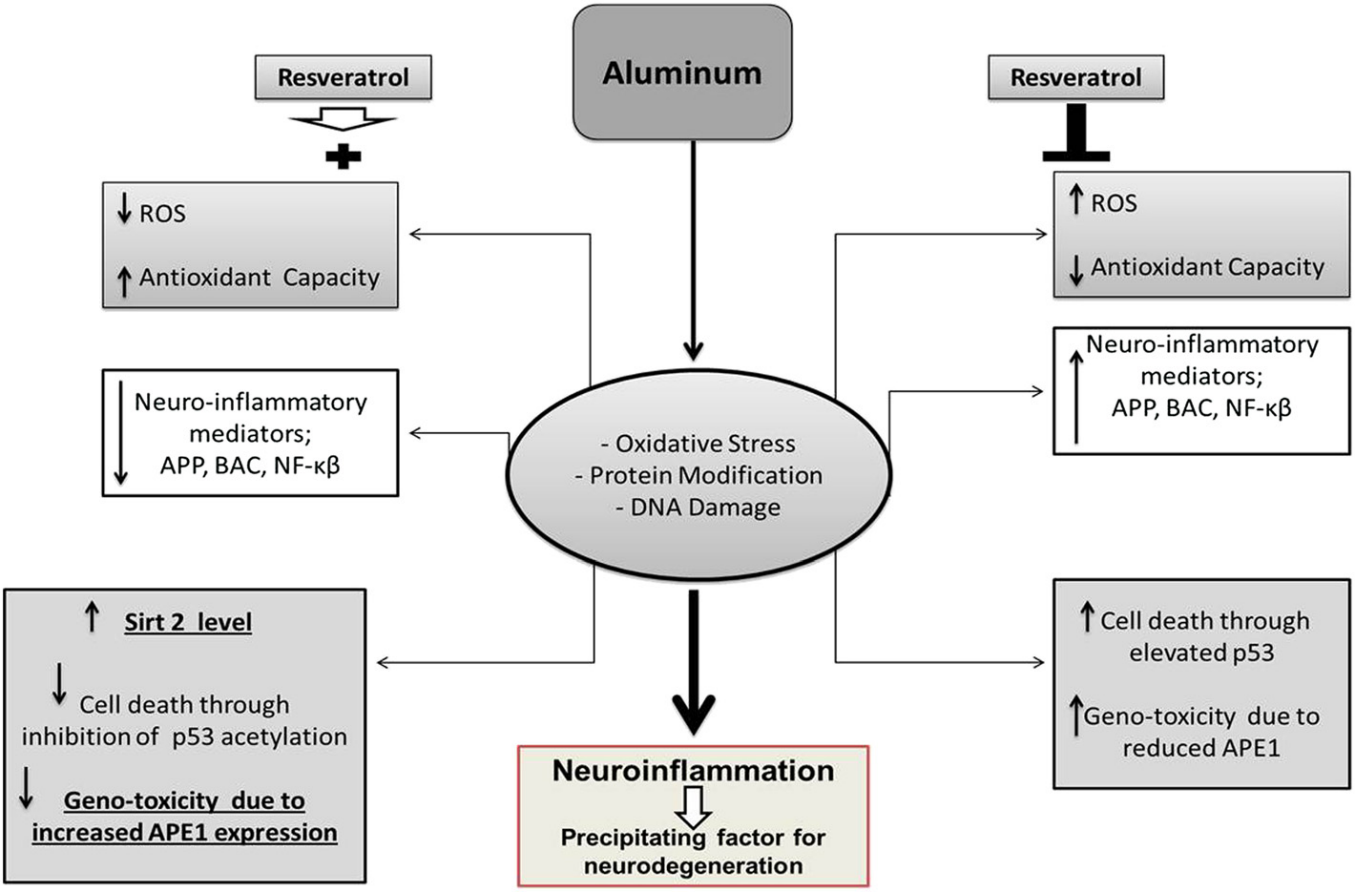
**Postulated mechanism of resveratrol protective effect against AlCl**_**3**_**–induced neurotoxicity.**

We have previously investigated APE1 in different experimental models in a trial to explore and highlight a new role during tissue fibrosis [45,46]. Consistently in the present study, by investigating APE1 expression in neuroinflammation model, the results revealed that AlCl_3_ significantly reduces both APE1 mRNA and protein levels. Furthermore, we show that in rats that did not receive any treatment post-lesion-induction and in self-recovery group, APE1 mRNA and protein levels started to re-elevate, which indicate that APE1 is essential during neuronal repair and regeneration. However APE1 level was also elevated in resv-treated group, but continuous revs administration seems to exert more protective effect on rat`s brain as indicated from the alterations in neuroinflammatory mediators expression which suggests that resv action is mediated, in part, by maintaining elevated APE1 level. Since it has been reported that p53 downregulates APE1 expression [47] and p53 transcriptional activity is modulated by sirt1 and sirt2 through deacetylation [42], we propose p53/APE1 signaling pathway as a novel resv-mediated target (Figure 8). We believe that understanding the molecular balance between total oxidant versus antioxidant capacities as well as pro-apoptotic versus pro-survival proteins during neuroinflammation is essential for therapeutics development.

## Conclusions

APE1/Ref-1 (APE1), a multifunctional protein possessing both DNA repair and transcriptional regulatory activities, has a pleiotropic role in controlling cellular response to oxidative stress such as exposure to neurotoxic agents. We have uncovered some important role of APE1 during neuroinflammation and consequently neurodegeneration pathogenesis. Our results suggest that the extent of inflammatory responses induced by AlCl_3_ in the main resident immunocompetent and phagocytic cells in CNS could be limited by resveratrol by maintaining high APE1 and may contribute positively to neuronal cell survival following exposure to cytotoxic agents.

## Competing interests

The authors declare that they have no competing interests.

## Authors' contributions

AZ and BM performed the experiments and analyzed the data. MM and KK provided useful advice and reviewed the manuscript. AZ and AB conceived the study, participated in its design and coordination, and wrote the manuscript. All authors read and approved the final manuscript.

## References

[B1] Pratico D Uryu K Sung S Tang S Trojanowski JQ Lee VM Aluminum modulates brain amyloidosis through oxidative stress in APP transgenic mice FASEB J 2002 16 1138 1140 12039845 10.1096/fj.02-0012fje

[B2] Izumi T Wiederhold LR Roy G Roy R Jaiswal A Bhakat KK Mitra S Hazra TK Mammalian DNA base excision repair proteins: their interactions and role inrepair of oxidative DNA damage Toxicology 2003 193 43 65 10.1016/S0300-483X(03)00289-0 14599767

[B3] Mitra S Izumi T Boldogh I Bhakat KK Hill JW Hazra TK Choreography of oxidative damage repair in mammalian genomes Free Radic Biol Med 2002 33 15 28 10.1016/S0891-5849(02)00819-5 12086678

[B4] Tell G Damante G Caldwell D Kelley MR The intracellular localization of APE1/Ref-1: more than a passive phenomenon? Antioxid Redox Signal 2005 7 367 384 10.1089/ars.2005.7.367 15706084

[B5] Okazaki T Chung U Nishishita T Ebisu S Usuda S Mishiro S Xanthoudakis S Igarashi T Ogata E A redox factor protein, ref1, is involved in negative gene regulation by extracellular calcium J Biol Chem 1994 269 27855 27862 7961715

[B6] Fujimura M Morita-Fujimura Y Narasimhan PJ Copin C Kawase M Chan PH Copper-zinc superoxide dismutase prevents the early decrease ofapurinic/apyrimidinic endonuclease and subsequent DNA fragmentation aftertransient focal cerebral ischemia in mice Stroke 1999 30 2408 2415 10.1161/01.STR.30.11.2408 10548678

[B7] Edwards M Kent TA Rea HC Wei J Quast M Izumi T Mitra S Perez-Polo JR "APE/Ref-1 responses to ischemia in rat brain" Neuroreport 1998 9 4015 4018 10.1097/00001756-199812210-00005 9926839

[B8] Gillardon F Bottiger B Hossmann KA Expression of nuclear redox factor ref-1 in the rat hippocampus following global ischemia induced by cardiac arrest Brain Res Mol Brain Res 1997 52 194 200 10 10.1016/S0169-328X(97)00237-4 9495540

[B9] Park KA Vasko MR Lipid mediators of sensitivity in sensory neurons Trends Pharmacol Sci 2005 26 571 577 10.1016/j.tips.2005.09.010 16185776

[B10] Mantha AK Dhiman M Taglialatela G Perez-Polo RJ Mitra S Proteomic study of amyloid beta (25–35) peptide exposure to neuronal cells: Impact on APE1/Ref1's protein-protein interaction J Neurosci Res 2012 90 1230 1239 10.1002/jnr.23018 22488727

[B11] Das DK Maulik N Resveratrol in cardio protection: a therapeutic promise ofalternative medicine Mol Interv 2006 6 36 47 10.1124/mi.6.1.7 16507749

[B12] Ates O Cayli SR Yucel N Altinoz E Kocak A Durak MA Turkoz Y Yologlu S Central nervous system protection by resveratrol in streptozotocin-induced diabetic rats J Clin Neurosci 2007 14 256 260 10.1016/j.jocn.2005.12.010 17258134

[B13] Karuppagounder SS Pinto JT Xu H Chen HL Beal MF Gibson GE Dietarysupplementation with resveratrol reduces plaque pathology in a transgenic model of Alzheimer's disease Neurochem Int 2009 54 111 118 10.1016/j.neuint.2008.10.008 19041676 PMC2892907

[B14] Aloisi F The role of microglia and astrocytes in CNS immune surveillance and immunopathology Adv Exp Med Biol 1999 468 123 133 10.1007/978-1-4615-4685-6_10 10635024

[B15] Chen Y Swanson RA Astrocytes and brain injury J Cereb Blood Flow Metab 2003 23 137 149 12571445 10.1097/01.WCB.0000044631.80210.3C

[B16] Gayle DA Ling Z Tong C Landers T Lipton JW Carvey PM Lipopolysaccharide (LPS)-induced dopamine cell loss in culture: roles of tumornecrosis factor-alpha, interleukin-1beta, and nitric oxide Brain Res Dev Brain Res 2002 133 27 35 10.1016/S0165-3806(01)00315-7 11850061

[B17] Qin L Liu Y Wang T Wei SJ Block ML Wilson B Liu B Hong JS NADPHoxidase mediates lipopolysaccharide-induced neurotoxicity and proinflammatory gene expression in activated microglia J Biol Chem 2004 279 1415 1421 14578353 10.1074/jbc.M307657200

[B18] Zeinstra E Wilczak N De Keyser J Reactive astrocytes in chronic active lesionsof multiple sclerosis express co-stimulatory molecules B7-1 and B7-2 J Neuroimmunol 2003 135 166 171 10.1016/S0165-5728(02)00462-9 12576238

[B19] Depino AM Earl C Kaczmarczyk E Ferrari C Besedovsky H del Rey A Pitossi FJ Oertel WH Microglial activation with atypical proinflammatory cytokine expression in a rat model of Parkinson's disease Eur J Neurosci 2003 18 27312742 10.1111/j.1460-9568.2003.03014.x 14656322

[B20] Itagaki S McGeer PL Akiyama H Zhu S Selkoe D Relationship of microglia and astrocytes to amyloid deposits of Alzheimer disease J Neuroimmunol 1989 24 173 182 10.1016/0165-5728(89)90115-X 2808689

[B21] Chen ZH Na HK Hurh YJ Surh YJ 4-Hydroxyestradiol induces oxidative stress and apoptosis in human mammary epithelial cells: possible protection by NFkappaB and ERK/MAPK Toxicol Appl Pharmacol 2005 208 46 56 10.1016/j.taap.2005.01.010 15901486

[B22] Yamamori T DeRicco J Naqvi A Hoffman TA Mattagajasingh I Kasuno K Jung SB Kim CS Irani K SIRT1 deacetylates APE1 and regulates cellular baseexcision repair Nucleic Acids Res 2010 38 832 845 10.1093/nar/gkp1039 19934257 PMC2817463

[B23] Ellman G Tissue sulfhydryl groups Arch Biochem Biophys 1959 82 70 77 10.1016/0003-9861(59)90090-6 13650640

[B24] Ohkawa H Ohishi N Yagi K Assay of peroxides in animal tissues by thiobarbituric acid reaction Anal Biochem 1979 95 351 358 10.1016/0003-2697(79)90738-3 36810

[B25] Habig W Pabst M Jacoby W Glutathione-S-Transferase the first enzymatic step in mercapturic acid formation J Biol Chem 1974 249 7130 7139 4436300

[B26] Aebi H Catalase in vitro Methods Enzymol 1984 105 121 126 11 6727660 10.1016/s0076-6879(84)05016-3

[B27] Reitman A Frankel S A colorimetric method for the determination of serum glutamic oxalacetic and glutamic pyruvic transaminases Am J Clin Path 1957 28 56 13458125 10.1093/ajcp/28.1.56

[B28] Chomczynski P Sacchi N Single-step method of RNA isolation by acid guanidiniumthiocyanate-phenol-chloroform extraction Anal Biochem 1987 162 156 159 2440339 10.1006/abio.1987.9999

[B29] Guo XM Tang RH Qin XY Yang J Chen GY "Effects of carbon disulfide on the expression and activity of nitric oxide synthase in rat hippocampus" Chin Med J (Engl) 2008 121 2553 2556 19187594

[B30] Livak KJ Schmittgen TD Analysis of relative gene expression data usingreal-time quantitative PCR and the 2(−Delta Delta C(T)) Method Methods 2001 25 402 10.1006/meth.2001.1262 11846609

[B31] Schreiber E Matthias P Müller M Schaffner W Rapid detection of octamer binding proteins with ‘mini-extracts’, prepared from a small number of cells Nuc Acid Res 1989 17 6419 10.1093/nar/17.15.6419 PMC318318 2771659

[B32] Burnette N Western blotting: electrophoretic transfer of proteins from sodiumdodecyl sulfate polyacrylamide gel to unmodified nitrocellulose and radiographicdetection with antibody and radioiodinated protein A Ana Biochem 1981 203 195 203 10.1016/0003-2697(81)90281-5 6266278

[B33] Smith MA Perry G Alzheimer disease: protein-protein interaction andoxidative stress Bol Estud Med Biol 1996 44 5 10 9369031

[B34] Wu Z Xu Q Zhang L Kong D Ma R Wang L Protective effect of resveratrol Against kainate-induced temporal lobe epilepsy in rats Neurochem Res 2009 34 1393 1400 10.1007/s11064-009-9920-0 19219549

[B35] Hayden MS Ghosh S Signaling to NF-kappaB Genes Dev 2004 18 21952224 10.1101/gad.1228704 15371334

[B36] Milne JC Lambert PD Schenk S Carney DP Smith JJ Gagne DJ Jin L Boss O Perni RB VCB Bemis JE Xie R Disch JS Ng PY Nunes JJ Lynch AV Yang H Galonek H Israelian K Choy W Iffland A Lavu S Medvedik O Sinclair DA Olefsky JM Jirousek MR Elliott PJ Westphal CH Small molecule activatorsof SIRT1 as therapeutics for the treatment of type 2 diabetes" Nature 2007 450 712 716 10.1038/nature06261 18046409 PMC2753457

[B37] Citron M B-secretase inhibition for the treatment of Alzheimer`s disease-promise and challenge Trends Pharmacol Sci 2004 25 92 97 10.1016/j.tips.2003.12.004 15102495

[B38] Kaether C Haass C Steiner H Assembly, trafficking and function of gammasecretase Neurodegener Dis 2006 3 275 283 10.1159/000095267 17047368

[B39] Jayadev S Case A Eastman AJ Nguyen H Pollak J Wiley JC Moller T Morrison RS Garden GA Presenilin 2 is the predominant gamma-secretase inmicroglia and modulates cytokine release PLoS One 2010 5 15743 10.1371/journal.pone.0015743 PMC3012089 21206757

[B40] Peck B Chen C-Y Ho K-K Fruscia PD Myatt SS Coombes RC Fuchter MJ Hsiao C-D Lam W-F SIRT Inhibitors Induce Cell Death and p53 Acetylation through Targeting Both SIRT1 and SIRT2 Mol Cancer Ther 2010 9 844 855 10.1158/1535-7163.MCT-09-0971 20371709

[B41] Tan Z Shi L Schreiber SS Differential Expression of Redox Factor-1 Associated with Beta-Amyloid-Mediated Neurotoxicity Open Neurosci J 2009 3 26 34 10.2174/1874082000903010026 19898678 PMC2773510

[B42] Ortega-Gutierrez S Molina-Holgado E Guaza C Effect of an andamide uptake inhibition in the production of nitric oxide and in the release of cytokines inastrocyte cultures Glia 2005 52 163 168 12 10.1002/glia.20229 15920730

[B43] Waetzig V Czeloth K Hidding U Mielke K Kanzow M Brecht S Goetz M Lucius R Herdegen T Hanisch UK c-Jun N-terminal kinases (JNKs) mediate proinflammatory actions of microglia Glia 2005 50 235 246 10.1002/glia.20173 15739188

[B44] Walton JR Aluminum Disruption of Calcium Homeostasis and Signal Transduction Resembles Change that Occurs in Aging and Alzheimer’s Disease J. Alzheimer Dis 2012 29 255 273 10.3233/JAD-2011-111712 22330830

[B45] Bassiouny AR Zaky AZ Abdulmalek SA Kandeel KM Ismail A Moftah M Modulation of AP-endonuclease1 levels associated with hepatic cirrhosis in rat model treated with human umbilical cord blood mononuclear stem cells Int J Clin Exp Pathol 2011 4 692 707 22076170 PMC3209610

[B46] Bassiouny AR Zaky A Fawky F Kandeel KM Alteration of AP-endonuclease1expression in curcumin-treated fibrotic rats Ann Hepatol 2011 10 516 530 21911894

[B47] Zaky A Busso C Izumi T Chattopadhyay R Bassiouny A Mitra S Bhakat KK Regulation of the human AP-endonuclease (APE1/Ref-1) expression by thetumor suppressor p53 in response to DNA damage Nucl Acids Res 2008 36 1555 1566 10.1093/nar/gkm1173 18208837 PMC2275136

